# Tissue Factor-Expressing Tumor Cells Can Bind to Immobilized Recombinant Tissue Factor Pathway Inhibitor under Static and Shear Conditions *In Vitro*

**DOI:** 10.1371/journal.pone.0123717

**Published:** 2015-04-07

**Authors:** Sara P. Y. Che, Christine DeLeonardis, Michael L. Shuler, Tracy Stokol

**Affiliations:** 1 Department of Biomedical Engineering, College of Engineering, Cornell University, Ithaca, NY, United States of America; 2 Department of Population Medicine and Diagnostic Sciences, College of Veterinary Medicine, Cornell University, Ithaca, NY, United States of America

## Abstract

Mammary tumors and malignant breast cancer cell lines over-express the coagulation factor, tissue factor (TF). High expression of TF is associated with a poor prognosis in breast cancer. Tissue factor pathway inhibitor (TFPI), the endogenous inhibitor of TF, is constitutively expressed on the endothelium. We hypothesized that TF-expressing tumor cells can bind to immobilized recombinant TFPI, leading to arrest of the tumor cells under shear *in vitro*. We evaluated the adhesion of breast cancer cells to immobilized TFPI under static and shear conditions (0.35 – 1.3 dyn/cm^2^). We found that high-TF-expressing breast cancer cells, MDA-MB-231 (with a TF density of 460,000/cell), but not low TF-expressing MCF-7 (with a TF density of 1,400/cell), adhered to recombinant TFPI, under static and shear conditions. Adhesion of MDA-MB-231 cells to TFPI required activated factor VII (FVIIa), but not FX, and was inhibited by a factor VIIa-blocking anti-TF antibody. Under shear, adhesion to TFPI was dependent on the TFPI-coating concentration, FVIIa concentration and shear stress, with no observed adhesion at shear stresses greater than 1.0 dyn/cm^2^. This is the first study showing that TF-expressing tumor cells can be captured by immobilized TFPI, a ligand constitutively expressed on the endothelium, under low shear *in vitro*. Based on our results, we hypothesize that TFPI could be a novel ligand mediating the arrest of TF-expressing tumor cells in high TFPI-expressing vessels under conditions of low shear during metastasis.

## Introduction

Tissue factor (TF), a 47kDa transmembrane protein, is constitutively expressed on the surface of fibroblasts and smooth muscle cells surrounding blood vessels [1]. The primary function of TF is to initiate coagulation upon vascular injury through binding to and acting as a cofactor for its enzymatic partner, factor VII. Previous studies have shown that TF is up-regulated and over-expressed in various types of cancer cells [2,3]. Over-expression of TF by tumor cells has been associated with paraneoplastic thrombosis [4–6]. Tissue factor has also been shown to have non-coagulant roles in cancer biology by promoting tumor proliferation, angiogenesis, and metastasis [1–3,5].

Cancer metastasis is a complex and poorly understood process involving multiple steps including invasion of tumor cells from the primary tumor, intravasation into the vasculature system, arrest and extravasation into surrounding tissue, and formation of a secondary tumor at established pre-metastatic niches [7–10]. Of these steps, TF has been shown to increase tumor cell invasion in extracellular matrices *in vitro* [11–13]. A recent study has also shown that TF is involved in the formation of the pre-metastatic niche [14]. Little is known on the role of TF in the later steps of the metastatic cascade or specifically if TF is involved in arrest of circulating tumor cells in blood vessels at sites of metastasis. Most studies on tumor cell adhesion to the endothelium have focused on classic adhesion receptor-ligand interactions (e.g. selectins and integrins), mimicking the recruitment of leukocytes during inflammation [15–17]. These studies have shown that selectins and integrins can mediate cancer cell adhesion to endothelium pre-activated by inflammatory cytokines. *In vivo* studies have suggested that non-classic interactions are involved in the adhesion of cancer cells to endothelial cells as rolling of cancer cell is not always observed prior to adhesion [18,19]. Instead, tumor cells simply arrest on unactivated endothelium in vessels of dimensions greater than that of the tumor cell, demonstrating that physical constriction was not the only cause of arrest.

Tissue factor pathway inhibitor (TFPI), the endogenous inhibitor of the TF-FVIIa complex, is constitutively expressed on the endothelium [20,21]. It inhibits the enzymatic activity of TF/FVIIa complex by binding to FVIIa and FXa through two Kunitz domains [22]. Since TFPI is constitutively expressed on the endothelium, and tumor cells over-express TF, we hypothesized that TF on tumor cells may bind to immobilized TFPI, thus providing *in vitro* support for a potential novel mechanism by which TF-expressing tumor cells could arrest on the endothelium under shear *in vivo*. Fischer *et al*. have shown that TF-expressing J82 bladder cancer cell lines adhered to recombinant TFPI under static conditions [23], but the interaction between TF-expressing tumor cells and TFPI under shear has not been investigated.

We found that, similar to J82 bladder tumor cells [23], high TF-expressing (MDA-MB-231), but not low TF-expressing (MCF-7), breast cancer cells bound to immobilized recombinant TFPI under static conditions in a FVIIa-dependent manner. Using a microfluidic device, we showed for the first time that high TF-expressing tumor cells also bound under low physiological shear to channels coated with immobilized recombinant TFPI. This binding and arrest of TF-expressing tumor cells to TFPI is dependent on the shear stress, coating concentration of TFPI, and FVIIa concentration. Based on our results, we hypothesize that endothelial cells with high constitutive TFPI expression could potentially arrest high TF-expressing tumor cells through adhesive interactions under low shear *in vivo*.

## Materials and Methods

### Reagents and antibodies

All reagents, unless noted, were from Sigma Aldrich (St Louis, MO). Human recombinant FVIIa and FX were purchased from Haematologic Technologies (Essex Junction, VT). Recombinant His-tagged human TFPI was purchased from R&D Systems (Minneapolis, MN). Protein G was purchased from EMD Millipore (Billerica, MA). Mouse anti-His, mouse IgG, and Alexa-488-conjugated secondary goat anti-mouse antibodies were purchased from Invitrogen (Carlsbad, CA). A mouse anti-TFPI antibody was purchased from Fitzgerald Industries International. Mouse monoclonal anti-TF antibodies (TF9-5B7 and TF9-10H10) were a generous gift from Dr. James Morrissey at University of Illinois.

### Cell lines and cell culture

We used MDA-MB-231 (a gift from Dr. Teresa Porri in NBTC, Cornell University; ATCC NCI-PBCF-HTB26, Mannasas, VA) as a model system for tumor cells expressing TF because the cell line expresses high amount of TF [24]. The MCF-7 (ATCC HTB-22, Mannasas, VA) breast cancer cell line with low expression of TF was used as a control. Both cell lines were cultured in DMEM (Life Technologies, Carlsbad, CA for MDA-MB-231, and Corning Cellgro, Mannasas, VA for MCF-7) completed with 10% (v/v) fetal bovine serum (Atlanta Biologicals, Norcross, GA) and 100U/ml penicillin-streptomyocin (Invitrogen, Carlsbad, CA). Human umbilical vein endothelial cells (HUVEC, Lonza, Basel, Switzerland) were cultured using EGM-2 media (Lonza, Basel, Switzerland). All cells were maintained in an incubator at 37°C and 5% CO_2._

### Cell preparation for adhesion experiments

Cells were serum-starved overnight to remove residual FVII or FX that may be present in serum-containing media. The cells were detached using an enzyme-free dissociation solution (Millipore, Billerica, MA), and then resuspended in serum-free media (with 2mM calcium and 5U/ml heparin to prevent non-specific binding of TFPI) at 1x10^6^ cells/ml. Cells were rested at room temperature for 30 minutes, during which time they were treated with antibodies (TF9-5B7, TF9-10H10 or an isotype control at 50μg/mL), if required. Cells that were to be incubated with TFPI were also pre-treated with FVIIa (10nM unless otherwise stated) with or without FX (10nM) for 10 minutes. For microfluidic experiments, cells were similarly prepared, but the resuspension medium also included 3% bovine serum albumin (BSA) to block non-specific tethering of cells to the surface.

### Ligand surface expression and ligand density

We used flow cytometry to verify surface expression of TF on the tumor cell lines, and TFPI on HUVEC. Cells were detached using an enzyme-free dissociation solution. Then, 5x10^5^ cells were resuspended in phosphate-buffered saline (PBS, Corning Cellgro, Mannasas, VA) with 1% BSA (PBSA) and cells were incubated with primary antibodies (80μg/mL for anti-TF antibody and 40μg/mL for anti-TFPI antibody or isotype IgG at similar concentrations) for 30 minutes on ice, and then washed with PBSA. An Alexa-488-conjugated secondary antibody (10μg/mL) was added to the cells for 30 minutes on ice, and then the cells were washed with PBSA. Cells were resuspended in PBS for flow cytometry (BD FACSCalibur). A total of 10,000 events were acquired and analyzed by frequency distribution curves of log fluorescent units.

To further determine the ligand density of TF on tumor cells and TFPI on HUVEC, we used a commercial kit (DAKO QIFIKIT, Carpinteria, CA) with flow cytometry as per manufacturer’s instructions. The results were analysed using FlowJo software v10 (Ashland, OR).

### Microfabrication of wells and microfluidic channels

**Fig 1:**
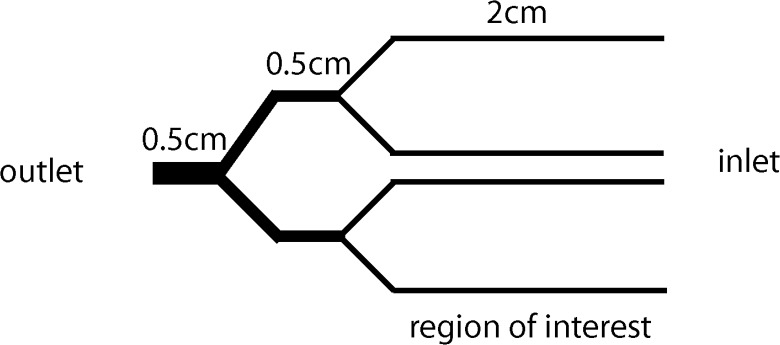
Schematic of microfluidic channel. The microfluidic channel consisted of four branches (120x120μm), which allowed for four simultaneous experiments under different coating conditions or cell treatments. The indicated region of interest (along the length of the 4 branches) is where adherent cells are quantified. Cell suspensions were introduced at the inlet and the outlet was connected to a syringe pump.

All microfabrication steps were performed at the Cornell NanoScale Science & Technology Facility. Poly-dimethylsiloxane (PDMS) was used to fabricate wells (0.8 x 0.8cm) for static adhesion and channels with four branches (120 x 120μm, Fig 1) for adhesion under shear. Mask designs were created using L-edit v16 (Tanner EDA, Monrovia, CA). To create the SU-8 masters for the wells and the channels, silicon wafers were spin-coated with SU-8 photoresist (MicroChem, Newton, MA) to create a film thickness of 200μm and 120μm respectively, following manufacturer’s instructions. The PDMS wells and channels were fabricated by pouring Sylgard 184 silicone elastomer kit (Dow Corning, Midland, MI; ratio of 10 base to 1 curing agent, w/w) over the SU-8 masters and curing in an oven at 60°C for 1.5 hours. Channels were plasma-cleaned and then sealed with glass slides.

### Characterization of surface density of immobilized TFPI

We used quartz crystal microbalance (QCM) to approximate the number of immobilized TFPI proteins in our *in vitro* system. This technique relates the frequency changes in the quartz crystal to the surface density of adsorbed or attached proteins (number/cm^2^) [25]. Quartz crystal sensors were coated with a thin layer of PDMS by spin-coating 1 drop of PDMS (1 curing agent: 10 base, diluted with 80% hexanes, w/w) at 6000RPM for 150 seconds [26]. The PDMS was cured at room temperature overnight. The measurements were performed and recorded using QCM200 (Stanford Research Systems, Sunnyvale, CA). The sensor was coated similarly to the microfluidic channels using 50μg/mL of Protein G, anti-His antibody, and TFPI in 3 separate incubation steps of 1 hour each, with a PBS wash between each incubation. The surface density was calculated based on the molecular weight of the proteins.

### Static adhesion

The PDMS wells were sterilized with 70% ethanol and then washed with PBS. Wells were then coated with proteins (10μg/mL fibronectin, 50μg/mL anti-TF IgG, isotype IgG or TFPI), incubated at 37°C for 1 hour, and then blocked with PBSA for 30 minutes at 37°C. Between steps, wells were washed with PBS. The wells were used immediately or stored at 4°C for use within 2 days of protein coating. Cells (5x10^4^) were added to the wells and incubated at 37°C for 1 hour. Non-adherent cells were removed by PBS washes. Half of the well (0.4 x 0.8cm) was imaged using bright field microscopy at low power (10x objective, Nikon Eclipse TE2000-U, Photometrics CoolSNAP HQ^2^ camera, Tucson, AZ). Adherent cells were counted at six pre-determined locations, and the count was normalized by the area of the field of view.

### Adhesion under shear

Channels were sterilized with 70% ethanol, then washed with deionized water and PBS. Each protein coating was performed at room temperature for 1 hour, and with PBS washes between steps. To properly orient the proteins, channels were first incubated with Protein G (100μg/mL), followed by antibodies (anti-TF IgG, isotype IgG or anti-His tag for TFPI coating at 100μg/mL, unless otherwise stated). Anti-His tag-coated channels were subsequently incubated with recombinant His-tagged TFPI (100μg/mL unless otherwise stated). All channels were blocked with 5% BSA for 30 minutes after protein coating.

Channels were then connected to a syringe pump (World Precision Instruments SP230IW, Sarasota, FL) and PBS was perfused through the channel at the experimental flow rate for 30 minutes to establish a stable flow profile. Cells (pre-treated with 10nM FVIIa and 10nM FX for TFPI-coated channels, unless otherwise indicated) were then introduced into the channels and monitored throughout the experiments in real-time using a motorized stage to observe behavior. Pictures were taken at pre-determined locations on the channel every 10 minutes for a total of 30 minutes to observe the change in cell adhesion over time. At the conclusion of the experiment, PBS was introduced for 10 minutes to remove non-adherent cells. Pictures were taken along the channel and the number of adherent cells was counted. The flow rate was then increased to a corresponding shear stress of 2.0dyn/cm^2^ for 10 minutes to remove loosely adherent cells. Repeat pictures were taken along the channel, and the number of adherent cells was quantified again and normalized by the area of the channel. Since there was a minimal difference in adherent cells before and after perfusion of PBS at the higher shear (indicating that few cells were loosely adherent), we only reported the final quantification.

### Calculation of shear

Shear stress along branched channels was calculated using hydraulic circuit analysis, in which branched channels can be modeled as hydraulic resistors in parallel to derive an equivalent circuit [27].

The average wall shear stress ($\bar{\tau }$) for a rectangular channel was calculated using an approximation given by Bahrami *et al*. (Eq 1), in which *A* is the area of the cross-section, *μ* is the dynamic viscosity, ε is the ratio of the channel height and width (ε<1), and $\bar{u}$ is the average velocity of the fluid, which can be derived from the pre-determined flow rate [28].

$$\bar{\tau }=\frac{6\mu \bar{u}}{\left[1\;-\;\frac{192}{\pi^{5}}\varepsilon \tanh\left(\frac{\pi }{2\varepsilon }\right)\right]\left(1+\varepsilon \right)\sqrt{A}\sqrt{\varepsilon }}$$ (1)

### Data analysis

Data was parametric and represented as mean ± standard deviation. Means of different cell lines were compared with an unpaired T-test. Means of the same cell line with different treatments were compared with ANOVA and Fisher post-test. All analyses were done using statistical software (Minitab 16, State College, PA) with significance set to p<0.05 (two tailed).

## Results

### TF is highly expressed on MDA-MB-231, but not on MCF-7, breast cancer cells

**Fig 2:**
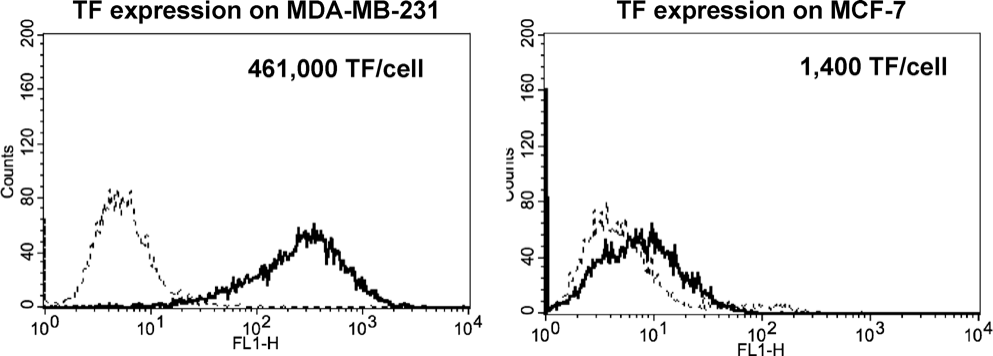
TF surface expression and density on breast cancer cells. Representative fluorescence histograms of TF expression on MDA-MB-231 and MCF-7 cells. Cells (5x10^5^) were incubated with a monoclonal antibody against TF (TF9-5B7, 80μg/mL), followed by an Alexa-488-conjugated secondary antibody (10μg/mL). Fluorescence was detected (bold line) using flow cytometry with isotype IgG as a control (dotted line). Tissue factor was strongly expressed on MDA-MB-231, but little expression was found on MCF-7 (n = 3). The surface ligand density is also shown for each cell line.

We verified surface expression of TF on the two breast cancer cell lines using flow cytometry, which showed a high surface expression of TF on MDA-MB-231, and a weak or no expression of TF on MCF-7 (Fig 2), as previously reported [24]. The TF ligand surface density was determined to be 461,000/cell for MDA-MB-231 and 1,400/cell for MCF-7.

### Quantification of amount of immobilized TFPI

Using QCM, we analyzed the surface density of immobilized recombinant TFPI molecules given by the coating protocol for the microfluidic channel. The QCM measurements qualitatively confirmed protein attachment onto the sensor surface, as the addition of each protein (Protein G, anti-His antibody and recombinant human TFPI) yielded a decrease in frequency, signifying an increase in mass and adhesion of the added proteins. Protein adsorption occurred rapidly as denoted by the immediate change in signal after protein addition, and the signal started to plateau within 30 minutes. Based on the mass of TFPI, the number of surface immobilized TFPI was calculated to be 3.4x10^12^ molecules/cm^2^. To determine how this amount of bound purified TFPI relates to constitutive expression on endothelial cells, we used HUVEC as our model endothelial cells, and measured their TFPI expression. Flow cytometry verified expression of TFPI by HUVEC, with ligand density quantified as 14,700/cell (S1 Fig). Assuming a diameter of 8μm for a cell, the expression of TFPI by HUVECs is approximately 3x10^10^ molecules/cm^2^, which is lower than that within the channel.

### MDA-MB-231 exhibits TF-specific adhesion to immobilized TFPI under static conditions

**Fig 3:**
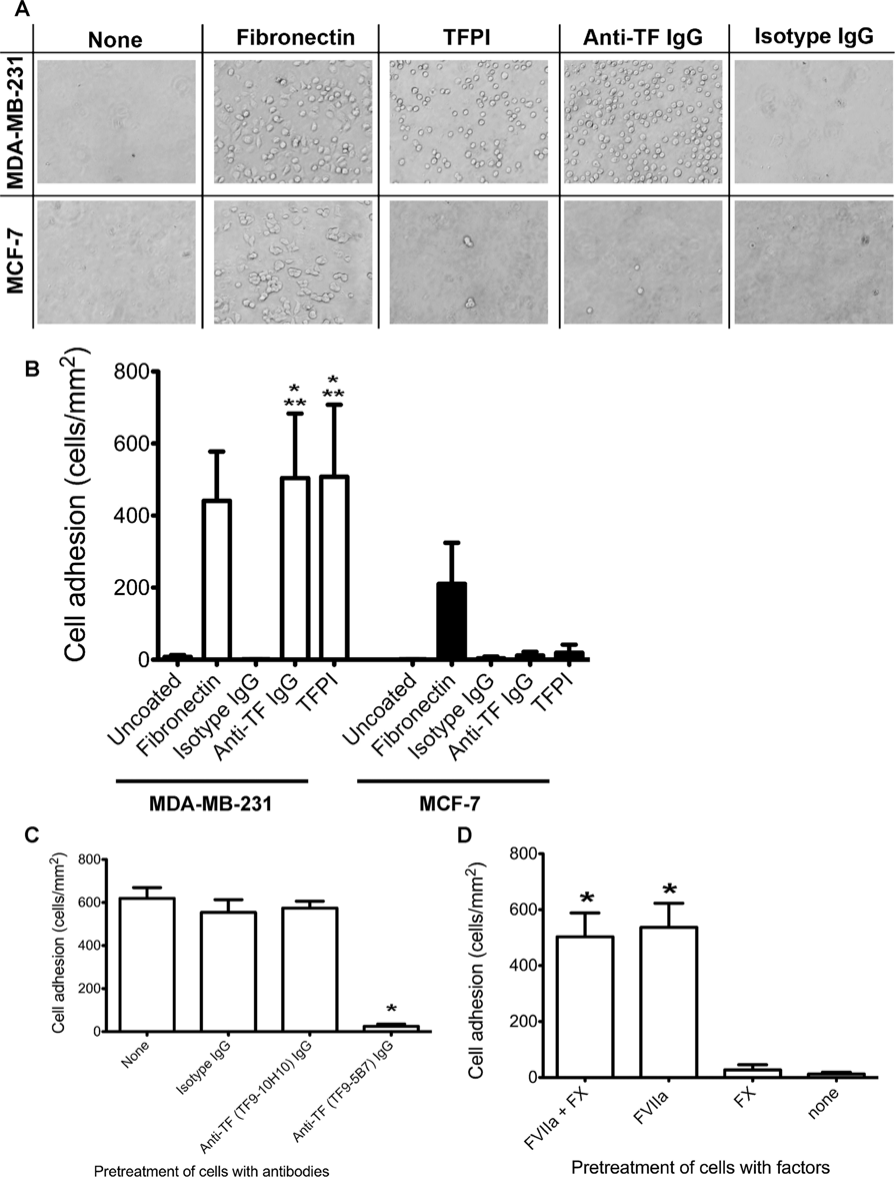
Static adhesion of tumor cells to protein-immobilized poly-dimethylsiloxane wells. MDA-MB-231 and MCF-7 (5x10^4^ cells) were incubated for 1 hour at 37°C in PDMS wells immobilized with TFPI (50μg/ml), using anti-TF IgG (TF9-5B7, 50μg/ml) as a positive control for TF-specific adhesion, fibronectin (10μg/ml) as a positive control for integrin adhesion, and uncoated or isotype IgG (50μg/ml) coated wells as negative controls. For TFPI-treated wells, cells were pretreated with 10nM FVIIa and 10nM FX for 10 minutes prior to addition to the wells. **A**. Representative bright field images of adherent cells on the different coatings. More MDA-MB-231 bound to TFPI- and anti-TF antibody-coated wells than MCF-7 cells. Both cell lines bound to fibronectin-coated wells. **B**. Adherent cells were counted and normalized by the area of the counted region (mean ± standard deviation). Significantly more MDA-MB-231 cells bound to TFPI- and anti-TF IgG-coated wells than MCF-7 (* p < 0.05, n = 6 for TFPI, n = 3 for anti-TF IgG). Significantly more MDA-MB-231 bound to TFPI- and anti-TF IgG-coated wells than uncoated or isotype IgG-coated wells (** p<0.05). **C**. MDA-MB-231 cells were pretreated with 50μg/ml anti-TF IgG (TF9-5B7 which blocks FVIIa binding to TF, and TF9-10H10 which does not block FVIIa binding to TF). The positive control had no antibody pretreatment, and isotype IgG pretreatment (50μg/ml) was used as a negative control. Blocking FVIIa binding to TF with the TF9-5B7 antibody significantly decreased adhesion to TFPI-coated wells compared to controls (* p < 0.05, n = 3). **D**. MDA-MB-231 cells were treated with different combinations of FVIIa (10nM) and FX (10nM) before incubation with TFPI-coated wells. Adhesion to TFPI was significantly decreased only when FVIIa was absent (* p < 0.05, n = 3).

After characterizing the tumor cells as a model system for TF-expressing cells, we proceeded to evaluate their adhesion to TFPI under static conditions. MDA-MB-231 cells bound to anti-TF antibody- and TFPI-coated wells, while MCF-7 only bound minimally to these TF-dependent substrates (Fig 3A and 3B). Both cell lines also bound to fibronectin via integrins as expected [29,30].

To confirm that the adhesion of MDA-MB-231 to TFPI was TF-dependent, we treated the cells with two different monoclonal anti-TF antibodies. TF9-5B7 is an antibody that binds to the FVII-binding domain of TF, which prevents binding to TFPI. TF9-10H10 binds to a non-FVII binding epitope and does not prevent binding of TF to TFPI [31]. Pre-treating MDA-MB-231 cells with TF9-5B7, but not TF9-10H10, significantly decreased adhesion to TFPI under static conditions (Fig 3C). We further demonstrated that only FVIIa, but not FX, was sufficient for adhesion of TF-expressing MDA-MB-231 to immobilized TFPI under static adhesions (Fig 3D). These results are similar to those reported by Fischer *et al*., who found TF/FVIIa-dependent adhesion of TF-expressing J82 bladder cancer cell lines to immobilized TFPI under static conditions [23].

### MDA-MB-231 exhibits FVIIa-specific adhesion to immobilized TFPI under low shear (0.35dyn/cm2)

Static adhesion does not fully recapitulate the interaction between circulating tumor cells and the endothelium *in vivo*. In circulation, tumor cells are under constant shear, limiting their interaction with expressed endothelial ligands. Using microfluidic devices, we are able to study the interaction between TF-expressing tumor cells and immobilized TFPI to determine if arrest of TF-expressing tumor cells to TFPI was possible under shear.

**Fig 4:**
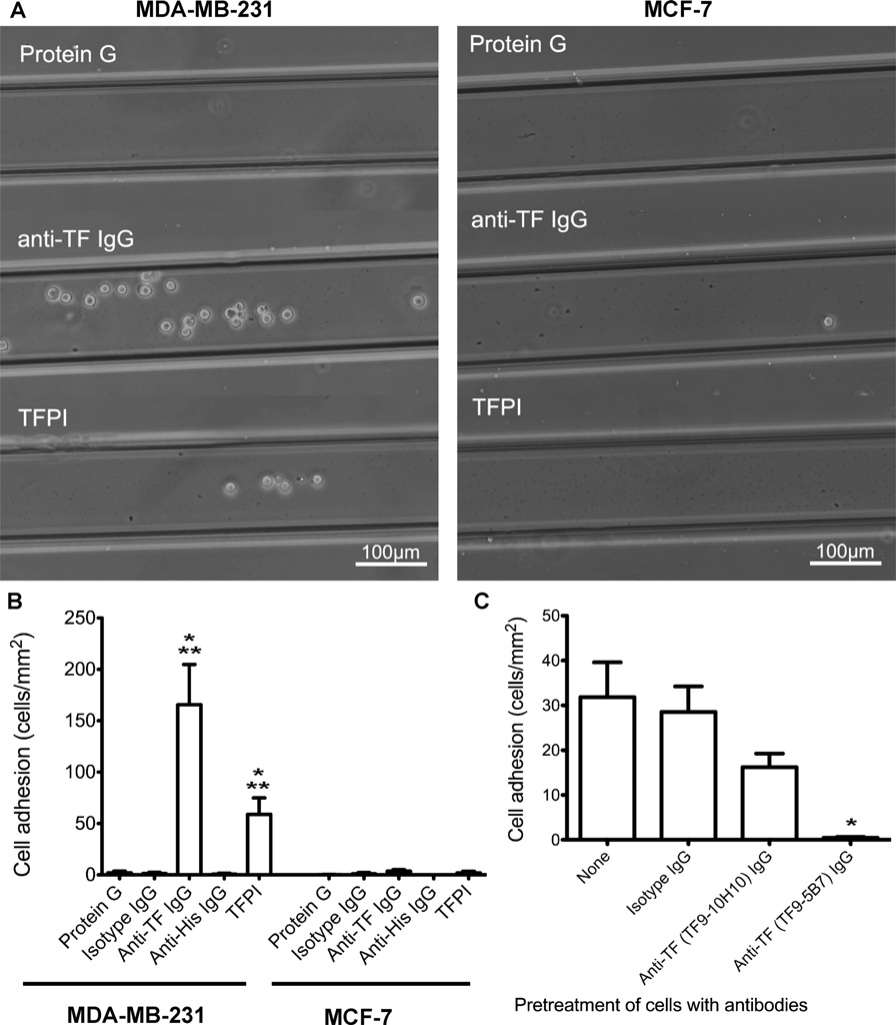
Adhesion of tumor cells to protein-immobilized microfluidic channels under low shear (0.35dyn/cm^2^). Microfluidic channels were incubated with Protein G (100μg/ml), then anti-TF IgG (100μg/ml), or an anti-His antibody (100μg/ml) followed by TFPI (100μg/ml). Isotype IgG (100μg/ml) and anti-His IgG (100μg/ml) antibodies were used as negative control for anti-TF IgG and TFPI respectively. Tumor cells (1x10^6^cells/mL, pre-treated with 10nM FVIIa and 10nM FX for TFPI-coated channels) were introduced into the channels at 0.35dyn/cm^2^ for 30 minutes, and non-specifically adhered cells were removed at 2.0dyn/cm^2^. The entire channel was imaged to quantify the number of adherent cells. **A.** Representative bright field images of adherent tumor cells on channels immobilized with Protein G (negative control), anti-TF IgG and TFPI showing that more MDA-MB-231 than MCF-7 cells were bound to both anti-TF IgG- and TFPI-coated channels. **B.** The number of adherent cells was counted and normalized by the channel area. MDA-MB-231 showed significantly higher adhesion to TFPI- and anti-TF IgG-coated channels than MCF-7 (* p < 0.05, n = 4 for anti-TF IgG, n = 3 for TFPI). Significantly more MDA-MB-231 bound to TFPI- and anti-TF IgG-coated channels than negative controls (** p<0.05). **C.** MDA-MB-231 cells were pretreated with 50μg/ml anti-TF IgG (TF9-5B7 which blocks FVIIa binding to TF, or TF9-10H10 which does not block FVIIa binding to TF). The positive control had no antibody pretreatment, and isotype IgG pretreatment (50μg/ml) was used as a negative control. Blocking FVIIa binding to TF with TF9-5B7 antibody significantly decreased adhesion to TFPI-coated channels (* p < 0.05, n = 4). The observed decrease in MDA-MB-231 adhesion with the TF9-10H10 antibody, albeit not significant with this stringent statistical test, could be due to steric hindrance of TFPI binding to the TF/FVIIa/FXa complex on the tumor cells.

Previous studies have shown that tumor cell adhesion occur under low shear through classic integrin-based interactions [10,17]. Hence, we first tested adhesion of high and low TF-expressing tumor cells to TFPI under low shear (0.35dyn/cm^2^). At this shear stress, we found that MDA-MB-231 strongly adhered to both anti-TF antibody- and TFPI-coated channels, while MCF-7 minimally bound (Fig 4A and 4B). Adhesion occurred quickly after the start of the experiment, and the number of adherent cells increased over time. No rolling behavior was observed with the tumor cells. Rather, the cancer cells would contact the immobilized TFPI and adhere immediately. We did not observe cell spreading, indicating a lack of integrin engagement and cell remodeling. We also observed that one adherent cell usually led to more adherent cells in its vicinity, suggesting mechanisms of secondary tethering and adhesion between tumor cells.

**Fig 5:**
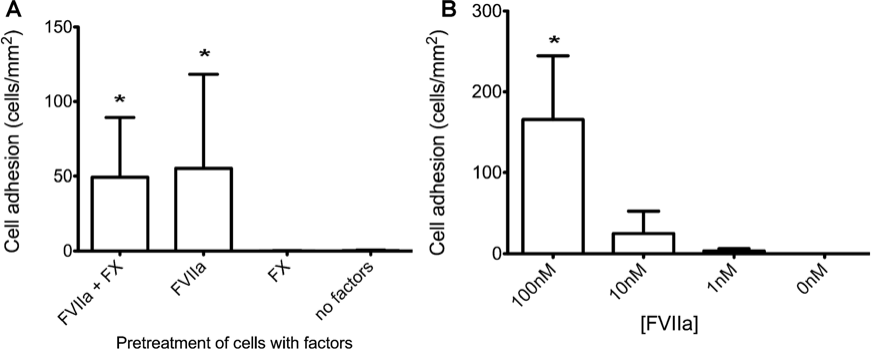
Effect of FVIIa and FX in adhesion of MDA-MB-231 to TFPI-immobilized channels (0.35dyn/cm^2^). **A.** MDA-MB-231 cells were treated with different combinations of FVIIa (10nM) and FX (10nM) before introduction into TFPI-immobilized channels. Adhesion of MDA-MB-231 to immobilized TFPI in microfluidic channels was abolished when FVIIa was absent (* p<0.05, n = 6). **B.** MDA-MB-231 cells were treated with different concentrations of FVIIa (0–100nM) prior to perfusion with TFPI-immobilized channels. Increasing the concentration of FVIIa to 100nM significantly increased adhesion of MDA-MB-231 to immobilized TFPI in microfluidic channels (* p < 0.05, n = 3).

When we pre-treated the MDA-MB-231 cells with a FVIIa-binding site blocking antibody (TF9-5B7), adhesion was markedly decreased (Fig 4C). We also showed that FVIIa (10nM) was the only requirement for TF-dependent adhesion of MDA-MB-231 to immobilized TFPI under this low shear stress (Fig 5A). These results indicate that adhesion of high TF-expressing MDA-MB-231 breast cancer cells to TFPI is mediated through FVIIa-bound TF, recapitulating our finding under static conditions.

Due to the high number of TF ligands expressed on the surface of MDA-MB-231 cells, we sought to determine if FVIIa concentration was a limiting factor for adhesion to TFPI under shear. With flow cytometry, we found that binding of FVIIa to MDA-MB-231 was saturated at 100nM of FVIIa (data not shown). Increasing the concentration of FVIIa from 10nM to 100nM significantly increased adhesion to TFPI at 0.35dyn/cm^2^ (Fig 5B), showing that FVIIa was indeed limiting the number of TF ligands on the tumor cells that are available to interact with TFPI under shear.

### Adhesion of MDA-MB-231 to immobilized TFPI is dependent on the concentration of protein coating, shear stress and concentration of FVIIa

**Fig 6:**
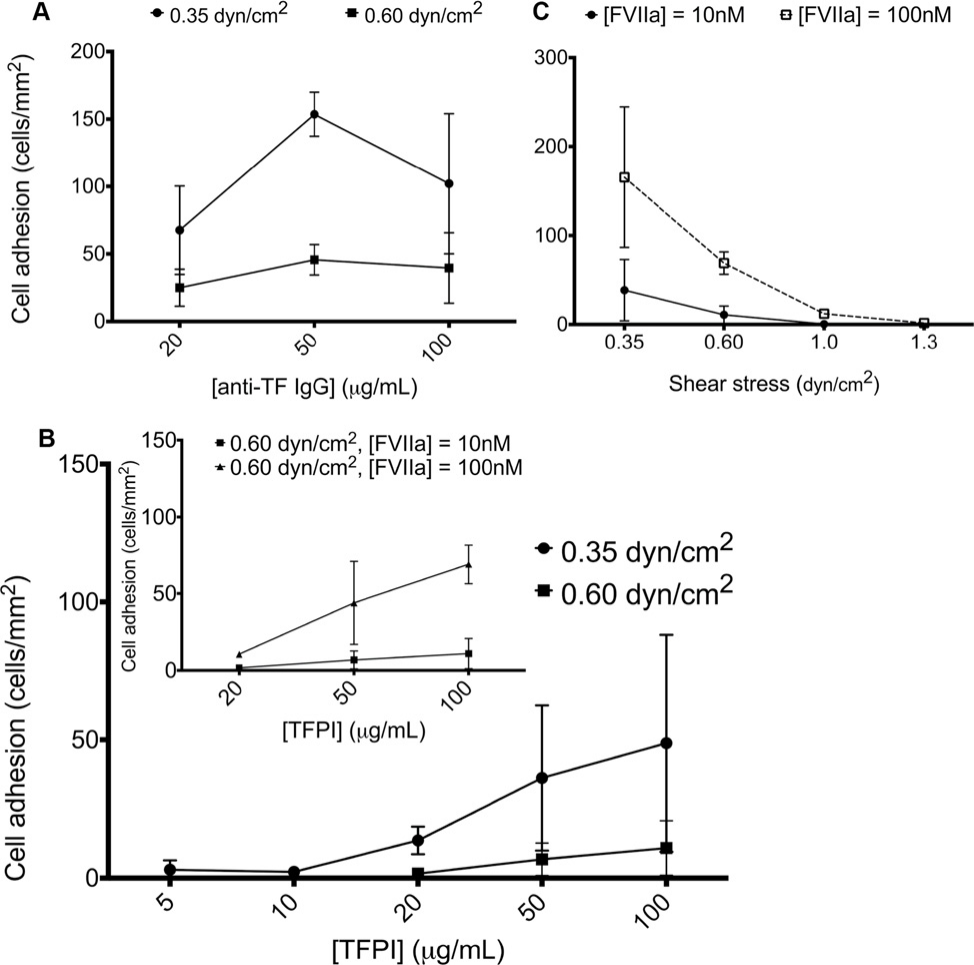
Effect of shear, TFPI-coating concentration and FVIIa concentration in MDA-MB-231 adhesion to protein-immobilized channels under shear. **A.** Microfluidic channels were immobilized with different concentrations of anti-TF IgG antibody (20–100μg/mL), and MDA-MB-231 cells were introduced at a shear of 0.35 and 0.60dyn/cm^2^. Adhesion of MDA-MB-231 cells to anti-TF IgG antibody reached a plateau at 50μg/mL at both shear stresses (n = 3). **B.** Microfluidic channels were immobilized with different concentrations of TFPI (5–100μg/mL), and MDA-MB-231 cells (pretreated with 10nM FVIIa and FX) were introduced at a shear of 0.35 and 0.60dyn/cm^2^. The adhesion of MDA-MB-231 increased with increasing TFPI concentration (n = 3). **Inset.** When FVIIa concentration was increased from 10nM to 100nM at a shear of 0.60dyn/cm^2^, the adhesion of MDA-MB-231 to TFPI-coated channels increased (n = 3). **C.** Microfluidic channels were immobilized with 100μg/mL TFPI, and MDA-MB-231 cells (pretreated with 10nM or 100nM FVIIa, and 10nM FX) were introduced at a range of shear stresses (0.35–1.3dyn/cm^2^). The adhesion of MDA-MB-231 decreased with increasing shear. Increasing the concentration of FVIIa from 10nM to 100nM increased adhesion of MDA-MB-231 to immobilized TFPI at 0.35 and 0.60dyn/cm^2^. A few tumor cells bound at 1.3dyn/cm^2^ with the higher, but not the lower, FVIIa concentration (n = 3).

Previous studies have shown concentration- and shear-dependent effects on antibody-based capture of tumor cells [32,33]. To determine if similar effects are involved in adhesion to immobilized TFPI, we first varied the concentration of the anti-TF antibody coating from 20μg/mL to 100μg/mL at 0.35dyn/cm^2^. We found that the adhesion of MDA-MB-231 was dependent on the concentration of immobilized anti-TF antibody, with a plateau in adhesion occurring at anti-TF antibody concentrations above 50μg/mL. We then increased the shear from 0.35dyn/cm^2^ to 0.60dyn/cm^2^ and found that adhesion of MDA-MB-231 was decreased at all concentrations of the anti-TF antibody coating at the higher shear (Fig 6A).

Next, we varied the concentration of TFPI from 5μg/mL to 100μg/mL. The observed adhesion of MDA-MB-231 in the channels was dependent on TFPI concentration, with minimal adhesion observed at concentrations of TFPI below 10μg/ml. Similar to our findings with the anti-TF antibody-coated channels, adhesion was decreased at all TFPI concentrations when the shear was increased from 0.35dyn/cm^2^ to 0.60dyn/cm^2^ (Fig 6B). In previous studies, antibody-based capture in straight channels generally was not possible above 0.5dyn/cm^2^ due to slow binding kinetics and short interaction time at higher shear [33]. Further modification to channel design (i.e. posts to affect streamlines to increase collision rates) or coating (i.e. use of selectins to induce rolling behavior) to enhance capture is often necessary at higher shear [34–36]. Nonetheless, in our system, we observed adhesion even at 0.60dyn/cm^2^, most likely an effect of the high TF ligand density expressed on MDA-MB-231.

Having already shown that FVIIa concentration affected adhesion of MDA-MB-231 under low shear (0.35dyn/cm^2^), we reasoned that not all TF sites were bound to FVIIa at 10nM and that adhesion under higher shear would be possible if we further increased the concentration of FVIIa. Hence, we saturated the TF on the surface of MDA-MB-231 cells by increasing FVIIa concentration from 10nM to 100nM under higher and more physiologically relevant shear. At 0.60dyn/cm^2^, we found that this saturating concentration of FVIIa did increase the adhesion of MDA-MB-231 to immobilized TFPI within the channels (Fig 6B inset). When the shear stress was further increased to 1.0dyn/cm^2^, we found that the saturating FVIIa concentration of 100nM permitted small amounts of adhesion whereas the use of signal-transducing concentrations of FVIIa (10nM) [37] did not. However, no adhesion was seen at the higher FVIIa concentration when the shear was further increased to 1.3dyn/cm^2^ (Fig 6C).

## Discussion

Tissue factor is abundantly expressed by malignant breast and other types of tumor cells, with expression correlating to metastatic potential [2,3]. The role of TF in tumor cell behavior has been examined, with prior studies primarily focusing on tumor-associated thrombosis, proliferation, and angiogenesis [1–3,5]. Fischer *et al*. evaluated TF as an adhesive ligand and showed that TF-expressing J82 bladder cancer cells bound to TFPI under static conditions and that TF was expressed at the leading edge of a tumor *in situ*, in close proximity to TFPI. Their results supported the role of TF-TFPI interactions in tumor invasion into the extracellular matrix [23]. To the best of our knowledge, there are no previous studies evaluating the arrest of TF-expressing tumor cells through interactions with TFPI under shear. In this study, we showed that high TF-expressing breast cancer cells could bind to immobilized TFPI *in vitro* under shear in a FVIIa- and TF-dependent manner. Our data lends support to our hypothesis that TF-TFPI may represent a novel receptor-ligand pair that could help mediate adhesion of circulating tumor cells under low shear in microvasculature at metastatic sites *in vivo*. If this hypothesis were correct, then the TF-TFPI interactions would likely be a contributory rather than the sole mechanism of mediating tumor cell adhesion to endothelial cells. Based on our data showing that high TF and TFPI concentrations are needed for adhesion to purified immobilized protein *in vitro*, these interactions would likely only be relevant for tumors that highly express TF [2] and vascular beds that highly express TFPI, such as the lung [38,39].

Factor VII was sufficient to mediate binding of high TF-expressing cancer cells to immobilized TFPI under static and low shear conditions, however the concentration of FVII was a limiting factor. Physiological concentrations of FVIIa (less than 1nM for coagulation and 10nM minimum for signaling [37]) was insufficient to saturate all of the TF expressed on MDA-MB-231. Increasing the FVIIa concentration to 100nM, (which saturated TF binding sites based on flow cytometric assessment) significantly increased the adhesion of cancer cells to immobilized TFPI in our study, particularly under shear conditions. Although the FVIIa concentration is higher than that normally present in plasma [37], high local concentrations are possible due to ectopic production of FVII by TF-expressing cancer cells [40–42].

In this study, we found that only FVIIa was necessary for the adhesion of TF-expressing MDA-MB-231 to TFPI, similar to results reported by Fischer *et al*. [23] Typically, TFPI forms a quaternary complex with TF, FVIIa and FXa by binding to FVIIa and FXa through the first and second Kunitz domain respectively [22]. The binding can occur by TFPI binding to FXa, and then to TF/FVIIa complex, or by TFPI binding to FVIIa and FX complexed to TF. However, FXa is not required for the binding of TF to TFPI, but rather TFPI can bind to the TF/FVIIa complex directly through its first Kunitz domain in the absence of FXa [43]. Instead, FXa only serves to strengthen the quaternary complex. This direct binding of TF/FVIIa to TFPI is in agreement with our result that FXa is not required in mediating adhesion of TF-expressing tumor cells to immobilized TFPI.

Most previous studies focus on capture of circulating tumor cells with selectin-mediated rolling, then subsequent integrin-mediated adhesion to endothelium, modeling the leukocyte adhesion cascade [10,15,44]. Our results support our current hypothesis that TFPI, which is constitutively expressed on the endothelium [20], can be another candidate responsible for helping the capture of high TF-expressing tumor cells to endothelial cells with high TFPI expression. We observed adhesion at shear stresses of 0.35dyn/cm^2^ to 1.3dyn/cm^2^, which overlaps with physiological shear (0.25dyn/cm^2^ to 4.0dyn/cm^2^ [45]) in capillary venules. Only a few cells adhered at 1.3dyn/cm^2^ when FVIIa and TFPI were saturating. The lack of adhesion above 1.0dyn/cm^2^ is not surprising, as a higher shear would decrease the time for interaction between TF and TFPI to induce adhesion. However, if selectins were upregulated on the endothelium (post-exposure to inflammatory cytokines or vasoactive mediators), it is possible that induction of rolling behavior in cancer cells may sufficiently slow the velocity of circulating tumor cells to promote adhesion to TFPI. Selectin-induced rolling by tumor cells has been well-characterized [15,46]. Unfortunately, MDA-MB-231 cells exhibit minimal selectin-mediated rolling [47] and we cannot use this cell line to test if selectin-mediated rolling would promote adhesion of tumor cells to immobilized TFPI in our *in vitro* system.

The microfluidic system used herein to study tumor cell adhesion is a simple system of straight channels and immobilized recombinant proteins, but it was sufficient to show that high TF-expressing tumor cells can bind to high concentrations of immobilized recombinant TFPI under low shear. Previous studies of the interactions between cells and proteins under shear mainly used parallel plate flow chamber or channels with dimensions approaching that of infinite planes [33,48]. The microfluidic channels in this study are more representative of the tumor vasculature in terms of dimensions. Green *et al*. have shown that rolling and adhesion of leukocytes are dependent on the gradient of shear across the cross section of the channel; increased adhesion was observed when the cross section is decreased to physiologically relevant dimensions [49]. Thus, the use of smaller dimensions may explain the observed binding at higher shear than previously reported.

There are no reports on the *in vivo* ligand density of TFPI on endothelial cells, so it was difficult to determine if the TFPI concentration in our *in vitro* system was within a physiological range. In QCM experiments, the concentration used was half of that in adhesion experiments with microfluidic channels due to volume constraints of the apparatus; however, the quick saturation in signal in QCM indicated that the amount added was sufficient to saturate the surface. We proceeded to estimate *in vivo* TFPI surface ligand density using HUVEC as a model endothelium. The TFPI concentration on these cells was calculated to be around 3x10^10^ proteins/cm^2^, which was 100-fold lower than the estimated density of our coated channel based on our QCM measurements. It should be noted that the measured QCM mass is a hydrated mass with contributions from both proteins and surrounding water molecules. As much as 90% of hydrated mass in QCM measurements may be due to water [50–52]. Thus, the actual concentration on the channel may be lower than what we estimated with QCM. Furthermore, the expression of TFPI also varies throughout the vasculature system depending on location and organ, with highest expressions in lung vasculature [38–39], one of the main sites of breast cancer metastasis. TFPI expression on endothelial cells is also increased in patients with cancer metastasis [53]. Thus, we speculate that it is possible that the high TFPI concentrations required to mediate adhesion under low shear in our microfluidic channels can occur in specific vascular beds that are relevant for metastasis *in vivo*, such as the lung. Our data also indicates that the surface density of TFPI on HUVECs is unlikely to support adhesion under shear conditions.

There is currently extensive interest in capturing circulating tumor cells using tumor-specific markers for diagnostic applications [32,34,36,54–56]. Usually, an antibody cocktail must be tailored for a specific cancer cell type based on its surface marker expressions, such as epithelial cell adhesion molecule (EpCAM) for epithelial cancers, human epithelial growth factor receptor 2 (HER2) for breast cancer and prostate specific membrane antigen (PSMA) for prostate cancer [32,34,55,56]. Tissue factor is over-expressed in different types of cancer [2] so it may be possible to use TF as a target to capture circulating tumor cells for an array of different tumors. The expression of TF is also correlated with the progression, malignancy and metastasis of cancer [57]. Tissue factor has been proposed as a potential marker for circulating breast cancer cells and stem cells, as well as a novel target for treatment of breast cancer [58–60]. Our study also provides data that supports the potential use of anti-TF antibodies to capture TF-expressing cancer or stem cells from the circulation.

In this study, we demonstrated that TF-expressing tumor cells bind to immobilized purified TFPI *in vitro* under low physiological shear. This data lends support to our hypothesis that TF-TFPI interactions represent a novel mechanism by which high TF-expressing tumor cells can arrest in high TFPI-expressing vasculature. The experimental results also illustrate that TF is a potential target for capturing circulating tumor cells.

## Supporting Information

S1 Fig Expression and density of TFPI on the surface of HUVEC. Representative flow cytometric fluorescence histogram of TFPI expression on HUVEC. Cells (5x10^5^) were incubated with a monoclonal antibody against TFPI (40μg/mL, bold line) or isotype control (40 μg/mL, dotted line), followed by an Alexa-488-conjugated secondary antibody (10μg/mL). The HUVEC did express TFPI with low to moderate surface density (n = 3).
(TIF)

## References

[1] Bluff JE, Brown NJ, Reed MWR, Staton CA (2008) Tissue factor, angiogenesis and tumour progression. BCR 10: 204–213. 10.1186/bcr1871 18373885 PMC2397518

[2] Rak J, Milsom C, Magnus N, Yu J (2009) Tissue factor in tumour progression. Best Pract Res Clin Haemato 22: 71–83. 10.1016/j.beha.2008.12.008 19285274

[3] Van den Berg YW, Osanto S, Reitsma PH, Versteeg HH (2012) The relationship between tissue factor and cancer progression: insights from bench and bedside. Blood 119: 924–932. 10.1182/blood-2011-06-317685 22065595

[4] Manly DA, Boles J, Mackman N (2011) Role of tissue factor in venous thrombosis. Annu Rev Physiol 73: 515–525. 10.1146/annurev-physiol-042210-121137 20690821 PMC3076951

[5] Rickles FR, Patierno S, Fernandez PM (2003) Tissue Factor, thrombin, and cancer. Chest 124: 58S–68S. 10.1378/chest.124.3 12970125

[6] Chu AJ (2011) Tissue factor, blood coagulation, and beyond: An overview. Int J Inflam 2011: Article ID 367284. 10.4061/2011/367284 PMC3176495 21941675

[7] Chambers AF, Groom AC, MacDonald IC (2002) Dissemination and growth of cancer cells in metastatic sites. Nat Rev Cancer 2: 563–572. 10.1038/nrc865 12154349

[8] Pantel K, Brakenhoff RH (2004) Dissecting the metastatic cascade. Nat Rev Cancer 4: 448–456. 10.1038/nrc1370 15170447

[9] Bacac M, Stamenkovic I (2008) Metastatic cancer cell. Annu Rev Pathol 3: 221–247. 10.1146/annurev.pathmechdis.3.121806.151523 18233952

[10] Wirtz D, Konstantopoulos K, Searson PC (2011) The physics of cancer: the role of physical interactions and mechanical forces in metastasis. Nat Rev Cancer 11: 512–522. 10.1038/nrc3080 21701513 PMC3262453

[11] Hjortoe GM, Petersen LC, Albrektsen T, Sorensen BB, Norby PL, Mandal SK et al (2004) Tissue factor-factor VIIa—specific up-regulation of IL-8 expression in MDA-MB-231 cells is mediated by PAR-2 and results in increased cell migration. Blood 103: 3029–3037. 10.1182/blood-2003-10-3417.Supported 15070680 PMC2837482

[12] Ma Z, Zhang T, Wang R, Cheng Z, Xu H, Li W et al (2011) Tissue factor-factor VIIa complex induces epithelial ovarian cancer cell invasion and metastasis through a monocytes-dependent mechanism. Int J Gynecol Cancer 21: 616–624. 10.1097/IGC.0b013e3182150e98 21543928

[13] Kakkar AK, Chinswangwatanakul V, Lemoine NR, Tebbutt S, Williamson RCN (1999) Role of tissue factor expression on tumour cell invasion and growth of experimental pancreatic adenocarcinoma. BJS 86: 890–894. 10.1046/j.1365-2168.1999.01153.x 10417560

[14] Gil-Bernabe AM, Ferjancic S, Tlalka M, Zhao L, Allen PD, Im JH et al (2012) Recruitment of monocytes/macrophages by tissue factor-mediated coagulation is essential for metastatic cell survival and premetastatic niche establishment in mice. Blood 119: 3164–3175. 10.1182/blood-2011-08-376426 22327225

[15] St. Hill CA (2011) Interactions between endothelial selectins and cancer cells regulate metastasis. Front Biosci 16: 3233–3251. 21622232 10.2741/3909

[16] Madsen CD, Sahai E (2010) Cancer dissemination-lessons from leukocytes. Dev Cell 19: 13–26. 10.1016/j.devcel.2010.06.013 20643347

[17] Bendas G, Borsig L (2012) Cancer cell adhesion and metastasis: selectins, integrins, and the inhibitory potential of heparins. Int J Cell Biol 2012 10.1155/2012/676731 PMC3296185 22505933

[18] Glinskii OV, Huxley VH, Turk JR, Deutscher SL, Quinn TP, Pienta KJ et al (2003) Continuous real time ex vivo epifluorescent video microscopy for the study of metastatic cancer cell interactions with microvascular endothelium. Clin Exp Metastasis 20: 451–458. 14524535 10.1023/a:1025449031136

[19] Haier J, Korb T, Hotz B, Spiegel H-U (2003) An intravital model to monitor steps of metastatic tumor cell adhesion within the hepatic microcirculation. J Gastrointest Surg 7: 507–515. 10.1016/S1091-255X(03)00023-4 12763408

[20] Ameri A, Kuppuswamy MN, Basu S, Bajaj SP (1992) Expression of tissue factor pathway inhibitor by cultured endothelial cells in response to inflammatory mediators. Blood 79: 3219–3226. 1596565

[21] Bajaj MS, Kuppuswamy MN, Saito H, Spitzer SG, Bajaj SP (1990) Cultured normal human hepatocytes do not synthesize lipoprotein-associated coagulation inhibitor: Evidence that endothelium is the principal site of its synthesis. Proc Natl Acad Sci 87: 8869–8873. 2247459 10.1073/pnas.87.22.8869 PMC55061

[22] Kato H (2008) Tissue factor pathway inhibitor: structure and function In: Tanaka K, Davie EW, Ikeda Y, Iwanaga S, Sito H, et al, editors. Recent Advances in Thrombosis and Hemostasis 2008. pp. 147–161.

[23] Fischer EG, Riewald M, Huang HY, Miyagi Y, Kubota Y, Mueller BM et al (1999) Tumor cell adhesion and migration supported by interaction of a receptor-protease complex with its inhibitor. J Clin Invest 104: 1213–1221. 10.1172/JCI7750 10545520 PMC409824

[24] Zhang X, Yu H, Lou JR, Zheng J, Zhu H, Popescu NI et al (2011) MicroRNA-19 (miR-19) regulates tissue factor expression in breast cancer cells. J Biol Chem 286: 1429–1435. 10.1074/jbc.M110.146530 21059650 PMC3020751

[25] Sauerbrey G (1959) The use of quartz oscillators for weighing thin layers and for microweighing. Z Phys 155: 206–222.

[26] Thangawng AL, Swartz M a, Glucksberg MR, Ruoff RS (2007) Bond-detach lithography: a method for micro/nanolithography by precision PDMS patterning. Small 3: 132–138. 10.1002/smll.200500418 17294484

[27] Kirby BJ (2010) Micro- and nanoscale fluid mechanics Transport in microfluidic devices. New York: Cambridge University Press.

[28] Bahrami M, Yovanovich MM, Culham JR (2005) Pressure drop of fully-developed, laminar flow in microchannels of arbitrary cross-section. Proceedings of ICMM 2005 3rd Internactional Conference on Microchannels and Minichannels. Vol. ICMM2005-7. pp. 269–280.

[29] Maity G, Choudhury PR, Sen T, Ganguly KK, Sil H, Chatterjee A. (2011) Culture of human breast cancer cell line (MDA-MB-231) on fibronectin-coated surface induces pro-matrix metalloproteinase-9 expression and activity. Tumour Biol 32: 129–138. 10.1007/s13277-010-0106-9 20821288

[30] Nista A, Leonetti C, Bernardini G, Mattioni M, Santoni A (1997) Functional role of alpha4beta1 and alpha5beta1 integrin fibronectin receptors expressed on adriamycin-resistant MCF-7 human mammary carcinoma cells. Int J Cancer 72: 133–141. 9212234 10.1002/(sici)1097-0215(19970703)72:1<133::aid-ijc19>3.0.co;2-k

[31] Morrissey JH, Fair DS, Edgington TS (1988) Monoclonal antibody analysis of purified and cell-associated tissue factor. Thromb Res 52: 247–261. 3194899 10.1016/0049-3848(88)90084-9

[32] Santana SM, Liu H, Bander NH, Gleghorn JP, Kirby BJ (2012) Immunocapture of prostate cancer cells by use of anti-PSMA antibodies in microdevices. Biomed Microdevices 14: 401–407. 10.1007/s10544-011-9616-5 22143878 PMC4074911

[33] Zheng X, Cheung LS-L, Schroeder JA, Jiang L, Zohar Y (2011) Cell receptor and surface ligand density effects on dynamic states of adhering circulating tumor cells. Lab on a chip 11: 3431–3439. 10.1039/c1lc20455f 21853194 PMC6765388

[34] Gleghorn JP, Pratt ED, Denning D, Liu H, Bander NH, Tagawa ST et al (2010) Capture of circulating tumor cells from whole blood of prostate cancer patients using geometrically enhanced differential immunocapture (GEDI) and a prostate-specific antibody. Lab Chip 10: 27–29. 10.1039/b917959c 20024046 PMC3031459

[35] Myung JH, Launiere C a, Eddington DT, Hong S (2010) Enhanced tumor cell isolation by a biomimetic combination of E-selectin and anti-EpCAM: implications for the effective separation of circulating tumor cells (CTCs). Langmuir 26: 8589–8596. 10.1021/la904678p 20155985 PMC2877147

[36] Nagrath S, Sequist L V, Maheswaran S, Bell DW, Irimia D, Ulkus L et al (2007) Isolation of rare circulating tumour cells in cancer patients by microchip technology. Nature 450: 1235–1239. 10.1038/nature06385 18097410 PMC3090667

[37] Rao LVM, Pendurthi UR (2005) Tissue factor-factor VIIa signaling. Arterioscler Thromb Vasc Biol 25: 47–56. 10.1161/01.ATV.0000151624.45775.13 15569823 PMC2838377

[38] Bajaj MS, Kuppuswamy MN, Manepalli AN, Bajaj SP (1999) Transcriptional expression of tissue factor pathway inhibitor, thrombomodulin and von Willebrand factor in normal human tissues. Thrombosis and haemostasis 82: 1047–1052. 10494762

[39] Osterud B, Bajaj MS, Bajaj SP (1995) Sites of tissue factr pathway inhibitor (TFPI) and tissue factor expression under physiologic and pathologic conditions. Thrombosis and haemostasis 73: 873–5. 7482419

[40] Koizume S, Miyagi Y (2010) Ectopic synthesis of coagulation factor VII in breast cancer cells: mechanisms, functional correlates, and potential for a new therapeutic target In: Gunduz E, editor. Breast Cancer—Current and Alternative Therapeutic Modalities. pp. 197–212.

[41] Yokota N, Koizume S, Miyagi E, Hirahara F, Nakamura Y, Kikuchi K et al (2009) Self-production of tissue factor-coagulation factor VII complex by ovarian cancer cells. British journal of cancer 101: 2023–2029. 10.1038/sj.bjc.6605406 19904262 PMC2795428

[42] Koizume S, Jin M-S, Miyagi E, Hirahara F, Nakamura Y, Piao JH et al (2006) Activation of cancer cell migration and invasion by ectopic synthesis of coagulation factor VII. Cancer research 66: 9453–9460. 10.1158/0008-5472.CAN-06-1803 17018600

[43] Callander NS, Rao LVM, Nordfanj O, Sandset PM, Warn-Cramer B, Rapaport SI. (1992) Mechanisms of binding of recombinant extrinsic pathway inhibitor (rEPI) to cultured cell surfaces. J Biol Chem 267: 876–882. 1730676

[44] Konstantopoulos K, Thomas SN (2009) Cancer cells in transit: the vascular interactions of tumor cells. Annu Rev Biomed Eng 11: 177–202. 10.1146/annurev-bioeng-061008-124949 19413512

[45] Lawrence MB (1990) Effects of Venous Shear Stress on CD18-Mediated Neutrophil Adhesion to Cultured Endothelium. Blood 75: 227–237. 1967215

[46] Orr FW, Wang HH, Lafrenie RM, Scherbarth S, Nance DM (2000) Interactions between cancer cells and the endothelium in metastasis. J Pathol 190: 310–329. 10.1002/(SICI)1096-9896(200002)190:3<310::AID-PATH525>3.0.CO;2-P 10685065

[47] Burdick MM, Henson KA, Delgadillo LF, Choi YE, Goetz DJ, Tees DF et al (2012) Expression of E-selectin ligands on circulating tumor cells: cross-regulation with cancer stem cell regulatory pathways? Front Oncol 2:103 10.3389/fonc.2012.00103 22934288 PMC3422812

[48] Giavazzi R, Foppolo M, Dossi R, Remuzzi A (1993) Rolling and adhesion of human tumor cells on vascular endothelium under physiological flow conditions. J Clin Invest 92: 3038–3044. 10.1172/JCI116928 7504697 PMC288509

[49] Green J V, Kniazeva T, Abedi M, Sokhey DS, Taslim ME, Murthy SK (2009) Effect of channel geometry on cell adhesion in microfluidic devices. Lab Chip 9: 677–685. 10.1039/b813516a 19224017

[50] Andreasson-Ochsner M, Romano G, Håkanson M, Smith ML, Leckband DE, Textor M et al (2011) Single cell 3-D platform to study ligand mobility in cell-cell contact. Lab on a chip 11: 2876–2883. 10.1039/c1lc20067d 21773619

[51] Liu Z (2010) Studies of biomacromolecule adsorption and activity at solid surfaces by surface plasmon resonance and quartz crystal microbalance with dissipation monitoring Virginia Tech.

[52] Höök F, Rudh M (2005) Quartz crystal microbalances (QCM) in biomacromolecular recognition. Review in BTi Molecular Biology Februrary/: 8–13. 15564302

[53] Inversen N, Lindahl AK, Abildgaard U (1998) Elevated TFPI in malignant disease: relation to cancer type and hypercoagulation. British Journal of Haematology 102: 889–895. 9734635 10.1046/j.1365-2141.1998.00875.x

[54] Stott SL, Hsu C-H, Tsukrov DI, Yu M, Miyamoto DT, Waltman BA et al (2010) Isolation of circulating tumor cells using a microvortex-generating herringbone-chip. Proc Natl Acad Sci 107: 18392–18397. 10.1073/pnas.1012539107 20930119 PMC2972993

[55] Thierry B, Kurkuri M, Shi JY, Lwin LEMP, Palms D (2010) Herceptin functionalized microfluidic polydimethylsiloxane devices for the capture of human epidermal growth factor receptor 2 positive circulating breast cancer cells. Biomicrofluidics 4: 32205 10.1063/1.3480573 21045921 PMC2967203

[56] Stott SL, Lee RJ, Nagrath S, Yu M, Miyamoto DT, Ulkus L et al (2010) Isolation and characterization of circulating tumor cells from patients with localized and metastatic prostate cancer. Sci Transl Med 2: 25ra23 10.1126/scitranslmed.3000403 20424012 PMC3141292

[57] Versteeg HH, Spek CA, Peppelenbosch MP, Richel DJ (2004) Tissue factor and cancer metastasis: the role of intracellular and extracellular signaling pathways. Mol Med 10: 6–11. 15502877 10.2119/2003-00047.versteeg PMC1431349

[58] Otero LL, Alonso DF, Castro M, Cinat G, Gabri MR, Gomez DE (2011) Tissue factor as a novel marker for detection of circulating cancer cells. Biomarkers 16: 58–64. 10.3109/1354750X.2010.533282 21128872

[59] Milsom C, Magnus N, Meehan B, Al-Nedawi K, Garnier D, et al (2009) Tissue factor and cancer stem cells: is there a linkage? Arterioscler Thromb Vasc Biol 29: 2005–2014. 10.1161/ATVBAHA.108.177444 19628788

[60] Cole M, Bromberg ME (2013) Tissue factor as a novel target for treatment of breast cancer. Oncologist 18: 14–18. 10.1634/theoncologist.2012-0322 23287882 PMC3556248

