# Supplementary figures and images for: Tissue Factor-Expressing Tumor Cells Can Bind to Immobilized Recombinant Tissue Factor Pathway Inhibitor under Static and Shear Conditions In Vitro
Source: PLoS One. 2015 Apr 7;10(4):e0123717. doi: 10.1371/journal.pone.0123717 (PMC4388665; doi:10.1371/journal.pone.0123717)

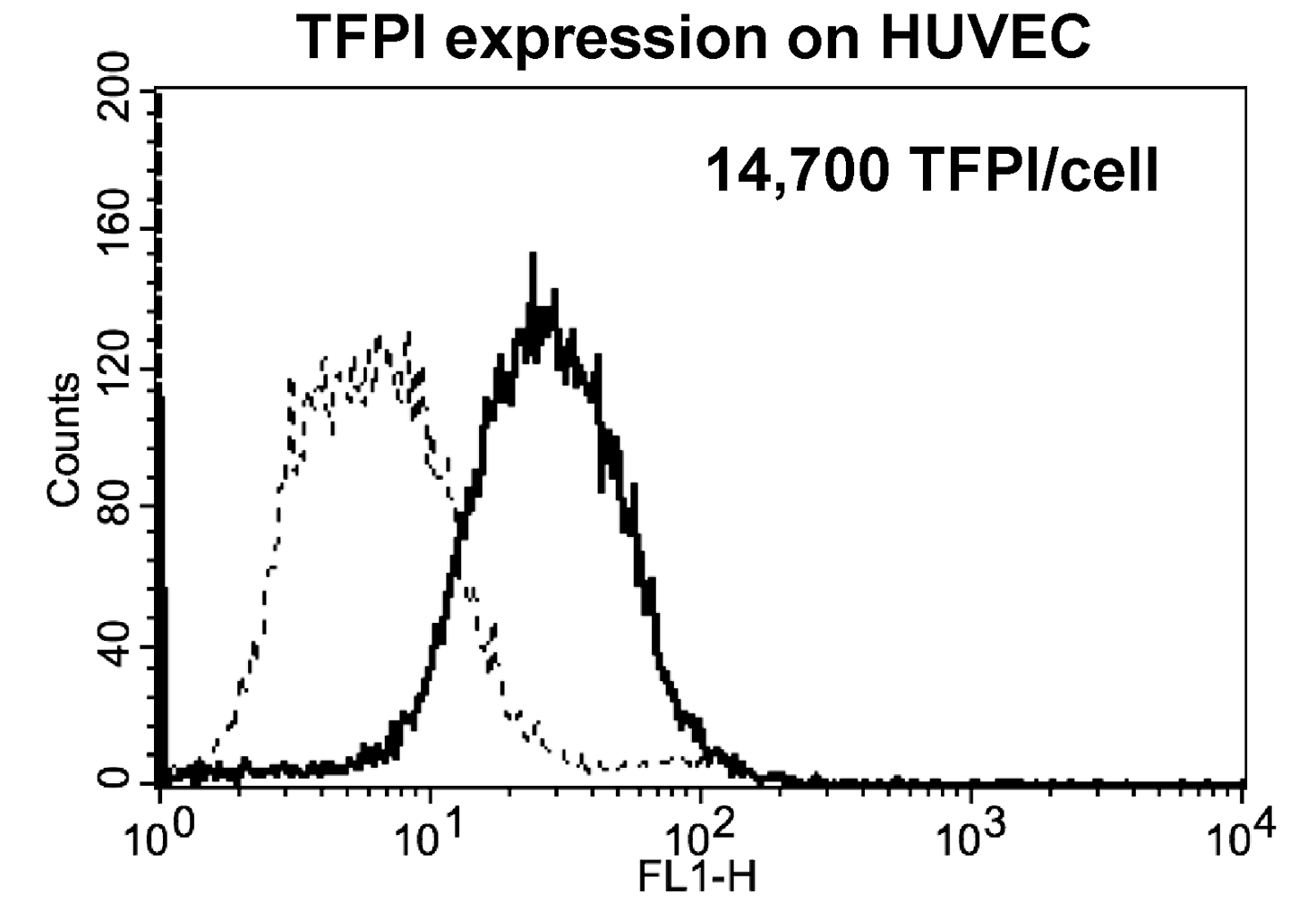

Supplement: S1 Fig — Representative flow cytometric fluorescence histogram of TFPI expression on HUVEC. Cells (5x10^5^) were incubated with a monoclonal antibody against TFPI (40μg/mL, bold line) or isotype control (40 μg/mL, dotted line), followed by an Alexa-488-conjugated secondary antibody (10μg/mL). The HUVEC did express TFPI with low to moderate surface density (n = 3). (TIF) [file pone.0123717.s001.tif]
